# Recent advances in drug repositioning and rediscovery for different therapeutic activities utilizing updated technological approaches

**DOI:** 10.1007/s11030-025-11248-w

**Published:** 2025-07-04

**Authors:** Eman S. Nossier, Manal M. Anwar, Mohamed Ayman El-Zahabi

**Affiliations:** 1 Pharmaceutical Medicinal Chemistry and Drug Design Department, Faculty of Pharmacy (Girls), Al-Azhar University, Cairo, 11754 Egypt; 2 The National Committee of Drugs, Academy of Scientific Research and Technology, Cairo, 11516 Egypt; 3 Department of Therapeutic Chemistry, Pharmaceutical and Drug Industries Research Institute, National Research Centre, El-Bohouth Street, P.O. Box 12622, Dokki, Cairo Egypt; 4 Pharmaceutical Medicinal Chemistry and Drug Design Department, Faculty of Pharmacy (Boys), Al-Azhar University, Cairo, 11884 Egypt

**Keywords:** Drug discovery, Drug repurposing, Activity-based experimental approaches, In silico-based computational approaches, Target-based screening

## Abstract

Traditional or de novo drug discovery is a time-consuming, costly, and high-investment process due to the high attrition rate. Therefore, many trials are conducted to reuse existing drugs to treat pressing conditions and diseases, since their safety profiles and pharmacokinetics are already available. Drug repurposing (DR) (also known as drug repositioning) is a strategy to identify a new indication of existing or already-approved drugs, beyond the scope of their original use. Various in silico-based computational and activity-based experimental approaches to incorporate available resources have been suggested for gaining a better understanding of disease mechanisms and the identification of repurposed drug candidates for personalized pharmacotherapy. This strategy is highly efficient, timesaving, low-cost, and minimum risk of failure. It maximizes the therapeutic value of a drug and consequently increases the success rate. This review introduced publicly available databases for drug repositioning and summarized the approaches taken for drug repositioning. Also, it highlighted and compared their characteristics, which should be addressed for the future realization of drug repositioning.

## Introduction

One tactic for discovering novel therapeutic applications of existing drugs with acceptable safety and pharmacokinetic characteristics is drug repurposing, also known as reprofiling. To address significant medical needs, particularly for long-term illnesses where developing new drugs is prohibitively expensive, drug repurposing—a complex area in drug discovery—has been investigated to identify potential new uses for existing drugs [1].

**Fig. 1:**
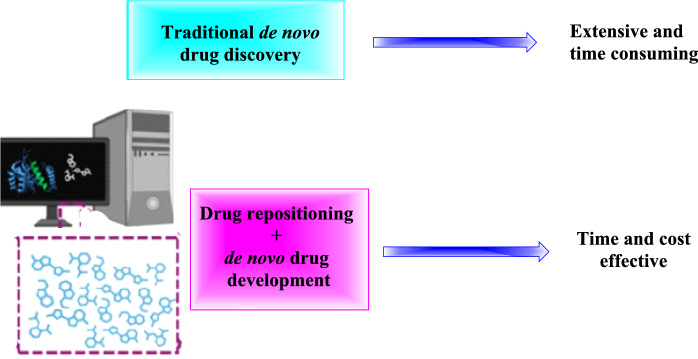
Combined application of traditional de novo drug development with drug repurposing improves the efficiency of the drug discovery process by reducing attrition rates and saving time

The conventional method of finding new drugs is costly and time-consuming. This process typically involves the discovery of compounds, preclinical testing, safety assessments, clinical investigations, FDA approval, and post-market surveillance. Drug repurposing, on the other hand, includes post-market safety monitoring, clinical studies, drug target analysis, and the evaluation of the approved drug library (Fig. 1) [2]. Furthermore, the rise of bioinformatics, artificial intelligence (AI), and computational biology is aiding in the precise identification of new pharmacological indications for already-approved drugs, which significantly accelerates the repurposing process [3–5]. Establishing the new indication based on existing expertise, such as pharmacokinetic and manufacturing data, greatly reduces drug development time and investment. The primary advantage of repurposed candidates is that preclinical models and early-stage human studies have often demonstrated sufficient safety. This means that, except for drug-disease interactions, they are less likely to fail subsequent efficacy trials due to safety concerns [6–8].

Licensed drugs have already undergone post-marketing monitoring, passed regulatory review, and undergone clinical testing. As only efficacy for the new indication should now be confirmed at the preclinical and clinical levels, much of the preclinical testing, safety assessment, and even phase I clinical trials may be avoided if dose compatibility is discovered (i.e., the required strength for the new indication is equal to or lower than the one used for the original indication) [9]. Most well-known and successful drug repurposing histories, including those involving sildenafil, minoxidil, and aspirin have emerged, often leveraging a medicine’s established pharmacology to address a clinical issue from a different field [10, 11]. However, in recent years, the drug discovery community has invested in using systematic, coordinated, data-driven approaches to drug repurposing, which typically incorporate computational support [10, 12, 13]. Lastly, this study will provide a more tangible understanding of how drug repurposing may aid in drug discovery and provide insights into some recently implemented tactics.

## Comparison between drug repurposing and conventional drug discovery

**Fig. 2:**
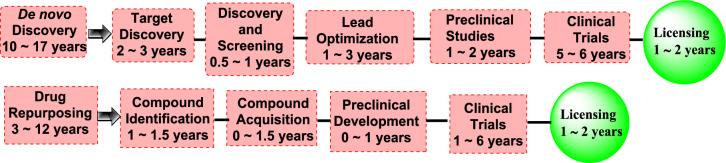
The average timing of each phase in a comparison between drug repositioning and typical de novo drug discovery

- The classical process to discover a new drug involves de novo investigation of novel molecules. This process includes five phases: discovery and preliminary studies, safety scrutiny, clinical trials, FDA evaluation, and FDA post-market safety monitoring. It is an expensive and time-consuming procedure that has a significant failure rate [14].
- Conversely, drug repositioning consists of just four phases: compound determination, molecular structure acquisition, development, and FDA post-market safety monitoring (Fig. 2) [15].

It is critical for the development of a novel pharmaceutical product for commercialization to perform preclinical studies to evaluate the drug’s efficacy and safety in laboratory settings, utilizing animal models. After that, to get the green light to examine it on human beings. The preclinical and clinical trials are the two main processes that comprise the in vitro and in vivo models needed to gather information on the biological and toxicological characteristics of the drug development process [16].

A. *Preclinical Trial:* This stage involves investigating the pharmacological impact of a novel chemical congener on animals in vivo. The medication then undergoes a clinical trial after passing the Investigational New Drug (IND) stage.
B. *Clinical Trial:* This step is further subdivided into 4 phases.
  *Phase I—safety:* Following ethics committee and regulatory approval, the first clinical investigation, or phase I study, is initiated. This phase represents the initial study conducted on human subjects. To determine whether the candidate behaves in the human body as preclinical studies have indicated, the candidate is typically tested on 20 to 80 healthy volunteers. At this stage, the primary focus is on evaluating the substance’s safety profile, or toxicity, in humans. Phase I involves assessing the drug’s absorption, its duration of action in the body, and establishing a safe dosage. It is important to note that women of childbearing age are generally excluded from phase I clinical trials due to safety concerns. Completing a phase I study can take up to a year.
  *Phase II—proof-of-concept:* Drug developers can request authorization to proceed to phase II clinical development if the phase I safety data are favorable. At this stage, the candidate is typically evaluated on a group of 100 to 300 individuals who have the condition they aim to treat. Here, safety and efficacy are assessed together as the drug’s minimum and maximum dosages are determined for use in the subsequent stage of development. Phase II can take up to two years on average.
  *Phase III—regulatory evidence:* Phase III is the next step if the safety and effectiveness data from phase II are positive. This stage is the final assessment of a medication before seeking market approval from pharmaceutical regulators. A phase III study typically enrolls at least 1000 participants, ensuring that sufficient data is collected to demonstrate the drug’s safety for humans and its expected therapeutic efficacy. Researchers document and report any adverse effects that individuals may experience during the phase III trial. This indicates that, to ensure accurate evaluation of those side effects, patients must be exposed to the medication for extended periods. Any adverse effects identified at this stage are subsequently included in the final product’s package leaflet.
  Phase III typically lasts between one and four years.
  **Market approval and launch**
  **The process of drug registration**
  An application for market approval, referred to as a Marketing Authorization Application (MAA) in the EU and a New Drug Application (NDA) or Biologics License Application (BLA) in the US, is submitted when phases I–III yield promising results. This application may include hundreds of thousands of pages of documentation summarizing all the data collected from the outset of the discovery phase, during which the primary investigator presents a case for FDA and EMA approval.
  It may take several months to prepare the application materials, and the authorities will require six to ten months to process the application.
  **Market launch**
  The candidate, or drug as it is now known, is prepared for market release if the regulatory authorities accept an application. The manufacturer and the prospective purchasers (government organizations or insurance firms, depending on the healthcare system) then start negotiating prices.
  *Phase IV:* Following a drug’s approval for sale, regulatory bodies may seek to follow up on phase IV research. To achieve this, data is collected from clinical practice or from actual care units that treat patients.
  Increasing pharmacovigilance is the goal. Additional safety tests are conducted, and phase IV investigations evaluate whether the medicine interacts with other substances. This step is particularly crucial for medications used to treat complex medical disorders or for pregnant patients who likely were not enrolled in phase I–III studies. Furthermore, phase IV studies may be relevant for medications designed to treat rare diseases, as phase I–III often involves a limited number of patients. Authorities are requesting further validation of the drug’s safety and effectiveness because the results of the earlier clinical studies have a lower level of statistical certainty.
  In general, it takes 12–15 years from substance discovery until a drug reaches the market.

- At the onset of a repositioning project, a range of preclinical (pharmacological, toxicological, etc.) and clinical efficacy and safety data are already available because the candidate drug has already undergone early stages of drug development, including structural optimization, preclinical and/or clinical trials, and has a clinical efficacy and safety profile. In this manner, the risks of early development failures—which are large in traditional approaches—are decreased, and costs are significantly decreased with the potential for improved clinical safety and a high success rate [17, 18].

**Fig. 3:**
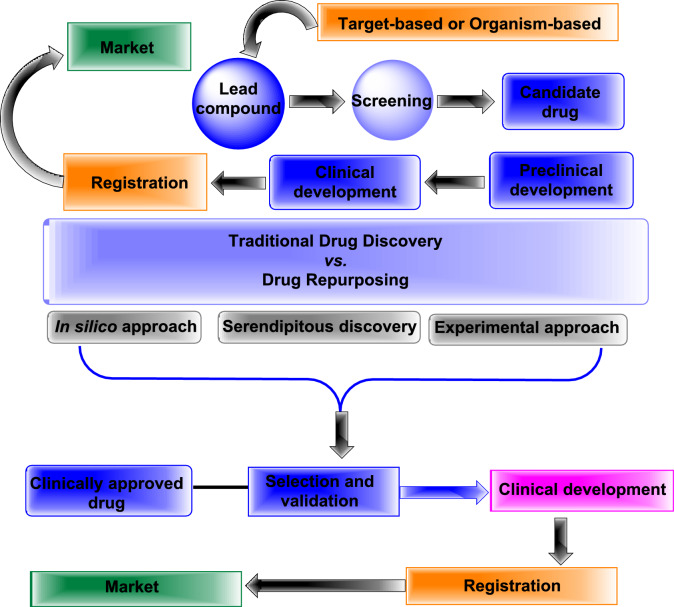
Traditional drug discovery *versus* drug repurposing and lead compound clinical development

Artificial intelligence (AI) technologies, structure-based drug design (SBDD), and in silico methodologies have significantly expedited the drug repurposing process in recent years (Fig. 3) [19, 20]. Drug repurposing can overcome current obstacles and open up new therapeutic opportunities when AI is integrated into drug discovery procedures in conjunction with in silico fields. These technologies have the potential to change the drug development landscape as they advance, which will ultimately result in improved therapies and better health outcomes for patients everywhere [20]. For instance, the oral anti-diabetic drug metformin (glucophage), which is commonly used to treat type 2 diabetes mellitus, has been developed as a cancer treatment and is presently undergoing phase II/phase III clinical studies [12, 21, 22].

**Fig. 4:**
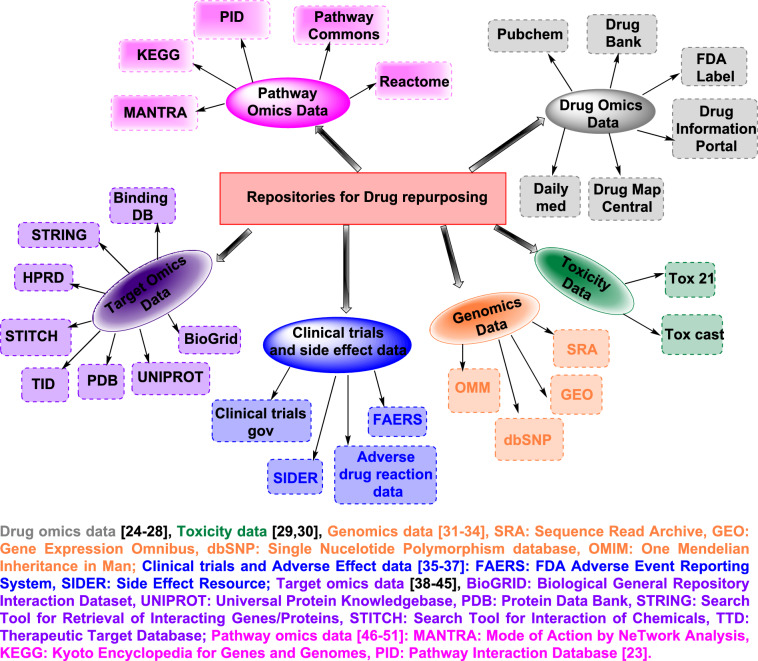
List of databases used in drug repurposing

- *The traditional discovery program* concentrates mainly on discovering medications to treat chronic and complex diseases, while the *drug repositioning* process targets the development medications for rapidly emerging and re-emerging infectious diseases, diseases that are difficult to treat, and neglected diseases.
- The availability of bioinformatics or cheminformatics approaches, large omics (proteomics, transcriptomics, metabolomics, genomics, etc.) data, and database resources allows for the use of disease-targeted-based repositioning methods to investigate unknown mechanisms of action for known or existing drugs (Fig. 4) [23]. These mechanisms include unknown drug targets, unknown drug–drug similarities, and new biomarkers for diseases, among other things.

## Classification of drug repurposing

### Based on its targets

#### On-target repurposing

On-target DR involves applying a medication molecule’s well-established pharmacological mechanism to a novel therapeutic indication. The condition is different with this approach, but the therapeutic molecule’s biological target is the same [12].

For instance, repositioning minoxidil (Rogaine), which operates on the same target and has two distinct therapeutic effects, results in an on-target profile. Originally, researchers developed minoxidil as an antihypertensive vasodilator medication. Because of its pharmacological effect as an antihypertensive vasodilator, which widens blood vessels and opens potassium channels to allow more oxygen, blood, and nutrients to reach the hair follicles, minoxidil treats male pattern baldness (androgenic alopecia) and is used to treat hair loss.

#### Off-target repurposing

The pharmacological mechanism in this case is initially unexpectedly but later investigated. The drug candidates target new targets outside of the original spectrum of treatment. As a result, both the signals and the targets are novel [21].

**Fig. 5:**
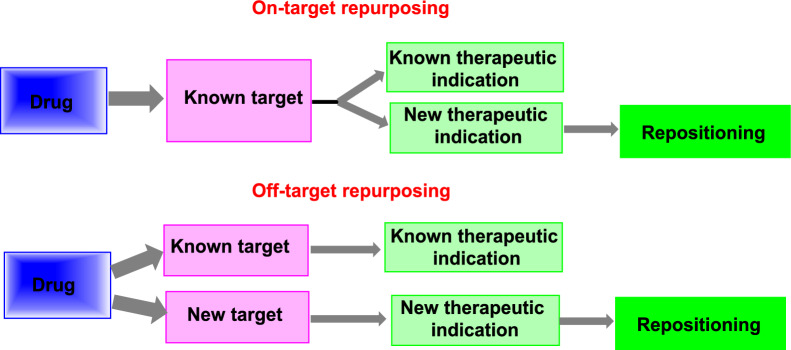
On- and off-target strategies of drug repositioning

 An excellent illustration of the off-target profile is aspirin (Colsprin). Historically, aspirin has been used as an NSAID to treat a variety of inflammatory and pain conditions. Additionally, it inhibits platelets’ natural function, which suppresses blood coagulation (clot formation) (antiplatelet medication). As a result, it is used to treat strokes and heart attacks. Aspirin has also been used in a novel way to treat prostate cancer (Fig. 5) [22].

### Based on its strategies

**Fig. 6:**
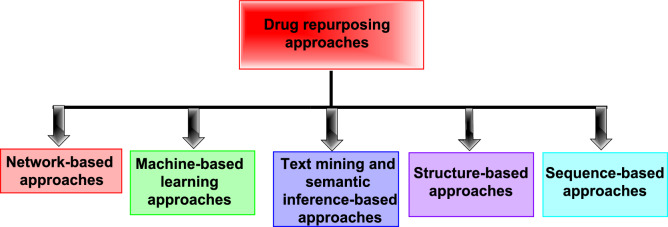
The current approaches of drug repositioning

According to the computational approach, Fig. 6 divides drug repurposing into: network-based, machine learning-based, text mining, and semantic inference-based methods. On the basis of data or information, this is further divided into three categories: sequence-based, structure-based, and signature-based techniques [52].

#### Network-based approaches

Computational drug repurposing frequently employs network-based methods due to their remarkable ability to integrate numerous connections and interactions among biological entities within biological networks [53]. Studies have shown that networks, including gene co-expression networks, drug-target proteins, drug-disease, drug–drug, and disease-disease interactions [54], as well as protein–protein networks, can aid in the identification of therapeutic biomarkers and drug targets, creating new avenues for drug development [55]. Network-based methods include DrugNet [56], Bi-Random walk [58], the restricted neighborhood search clustering algorithm (RNSC) [57], and the prioritization of illness genes and the inferring of protein complex relationships (PRINCE) [54].

#### Machine-based learning approaches

Machine learning (ML) methods for drug repurposing utilize algorithms to evaluate vast amounts of biological, chemical, and clinical data, identifying existing medications that could be repurposed for new therapeutic indications. These methods frequently use drug similarity analysis, in which algorithms look for medications with comparable qualities that might be repurposed by comparing molecular fingerprints or chemical structures. Furthermore, biological data analysis is essential; to identify links between medications and disease, machine learning algorithms look at gene expression profiles, protein interactions, and disease pathways [59].

#### Text mining and semantic inference-based drug repurposing approaches

One tool for retrieving possible data that helps researchers find novel medications is literature or text mining [60]. Extensive scientific literature can reveal many hidden indirect relationships between drug-target disorders. Medical Subject Heading (MeSH) phrases are used in literature mining to find pertinent information [61, 62]. The most common resources for drug repurposing based on text-mining methodologies are EDGAR (Extraction of drugs, genes, and relations) [63], FACTA+ (https://www.nactem.ac.uk/facta/), Biovista (https://www.biovista.com/), and DrugQuest (http://cbg.med.uoc.gr/).

Text mining refers to the automated extraction of essential information from unstructured text data, such as scientific articles (e.g., PubMed abstracts), patents, and clinical trial reports. However, semantic inference goes beyond basic keyword searches. It involves understanding the meaning and connections between concepts in the text. This capability allows for the identification of indirect or hidden relationships among biological targets, drugs, and diseases [14].

#### Structure-based drug repurposing approaches

Structure-based drug repurposing identifies new uses for existing pharmaceuticals by utilizing the shapes of molecules. Certain medications may have a high affinity for other target proteins that cause various diseases in addition to their original therapeutic targets [64]. A technique known as molecular docking is frequently used in the structure-based approach to predict how well a medication will fit into its probable target proteins. To evaluate the strength of a drug’s potential binding to its target proteins, scientists currently employ a variety of software programs, including AutoDock [65], AutoDock Vina [66], SwissDock [67], FlexX [68], Surflex [69], CDOCKER [70], and GLIDE [71]. To confirm the docking data, scientists frequently employ extra techniques like molecular dynamics modeling [72]. Notable accomplishments include mecamylamine, which was first used as an antihypertensive but was later repurposed as a nicotinic receptor antagonist for depression [74], and ropinirole, which was first used for hypertension but was later repurposed as a D2 receptor agonist for Parkinson’s disease [73].

#### Sequence-based drug repurposing approaches

Employing this method, researchers look for similarities in the amino acid sequences of proteins. A medication may also help treat additional illnesses if it can affect a protein that shares a similar sequence to its original target [75]. Established medications like ribavirin, simeprevir, and danoprevir have recently demonstrated the potential of sequence-based drug repurposing by treating COVID-19.

#### Signature-based drug repurposing approaches

Using patterns in gene or protein expression (signatures) from disease-related omics data, signature-based drug repurposing techniques look for novel applications for already-approved medications [76]. If a medication can return the impacted gene’s expression levels to normal, it may help treat the illness. For instance, researchers commonly combine the connectivity map (CMap) database with the expression databases of the Gene Expression Omnibus (GEO) [77] and The Cancer Genome Atlas (TCGA) [78]. Signature-based drug repurposing has clear advantages over other drug repurposing techniques because of its accuracy and focused approach [79].

**Fig. 7:**
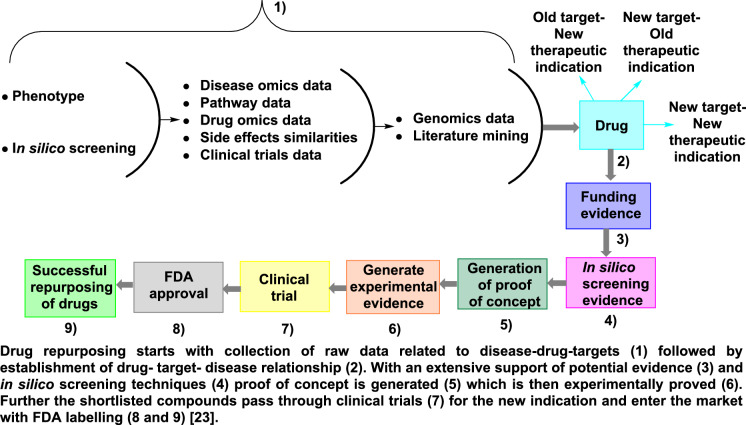
The different stages of drug repurposing

Based on prior strategies, it is possible to summarize that drug repurposing occurs in two stages (Fig. 7) [23]:

- *The first stage*: In silico screening of approved or marketed drugs against a particular therapeutic target, and the shortlisted are further processed for investigation in specific pathophysiological pathways of the disease of interest using in vitro and in vivo methodologies.
- *The second stage* is to enter the clinical trials for the respective indication. Drugs may be repurposed at any stage of their evolution right from their discovery.

## Difficulties with the repurposing process

- Optimizing inclusion and exclusion criteria in target population selection is crucial. The primary responsibility for assessing the drug’s anticipated effect is choosing treatment groups. Inadequate subject selection may lead to unintended adverse effects rather than therapeutic benefits [80].
- Timely achievement: When repurposing an old medication for a new indication, many factors must be taken into account, such as the dosage schedule and administration method, to achieve a notable benefit for the new indication. One obstacle is to optimize the formulation without making the medicine unstable [81].
- Repurposing a medicine to target a different patient group with a distinct set of physiological parameters may result in unanticipated adverse outcomes, necessitating a thorough examination of each reaction.
- Prerequisite information on drug–drug interactions, pharmacodynamics, and pharmacokinetics of the drugs, with a focus on toxicity profile, is necessary for repurposing medication combinations.

By solving issues in repurposing across regulatory agencies, industry, and academia, the area of drug discovery can attain unprecedented levels of efficiency, accuracy, and impact through the use of AI capabilities. This collaborative model accelerates discovery while ensuring accessible, effective, and safe medications for patients globally [20].

## Uses of repurposing drugs

This review discussed recent developments in the field of drug repositioning with the two major public health challenges on a global scale, infectious illnesses and cancer, in addition to other cases of successful drug repurposing [82, 83].

### Treatment of infectious diseases

#### Antileishmanial agents

According to recent research, macrophages’ increased synthesis of prostaglandin E2 (PGE2) plays a significant role in Leishmania’s ability to evade the host immune system and proliferate throughout the body [84–86]. Simultaneously, fumarate reductase was discovered to be crucial for Leishmanian energy production, but not for host cells [87, 88]. So, small molecules with multiple functions that stop macrophages and *Leishmania donovani* fumarate reductase from making PGE2 may be better at stopping visceral leishmaniasis infections.

According to reports, sulfuretin **1** and its analogs have the ability to both directly inhibit *L. donovani* fumarate reductase and suppress the generation of PGE2 by macrophages [89]. A hit chemical compound with promising action against *L. donovani* was identified as a C^6^-methoxy derivative of sulfuretin **2**. However, one significant limiting factor impeding progress for additional research is the poor water solubility of these analogs. It is necessary to alleviate these compounds’ poor solubility by adding polar moieties.

Glycosides that dissolve readily in water, such as bractein **3**, an *O*^*4*^-glycoside (ring-B) of a sulfuretin analog, can be broken down in the digestive system [90, 91]. Additionally, bractein’s antileishmanial action was negatively impacted by *O*^*4*^-glycosidation. To increase water solubility and sustain or improve strong antileishmanial activity, an alternative approach is therefore required.

Using a sulfuretin analog with a 2′,5′-dimethoxy substitution at ring A, pyrrolidine **4** demonstrated exceptional potency against *L. donovani* promastigotes, with an IC_50_ value of 4.1 μM when compared to edelfosine as a standard drug (IC_50_ = 3.9 μM).

**Fig. 8:**
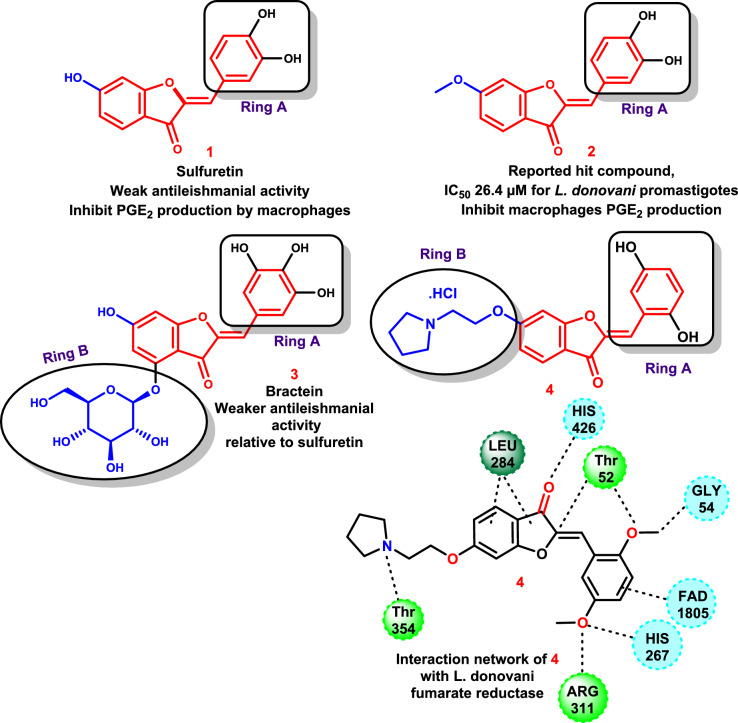
The molecular structures of the drugs sulfuretin and bractein and the predicted binding mode of the hit compound **4** with *L. donovani* NADH-dependent fumarate reductase

Moreover, inserting *O*^*6*^-aminoalkyl moieties to sulfuretin analogs as a 2-(pyrrolidin-1-yl)ethoxy fragment in **4** enhanced its ability to dissolve in water and produced a boost in inhibitory effectiveness against *L. donovani* promastigotes that was comparable with the reference’s potency. Compound** 4** exhibited favorable interactions with the amino acid residues Lys50, Thr52, Gly54, His267, Leu284, Arg311, Tyr354, and His426 through both hydrophilic and hydrophobic interactions. The compound’s anticipated binding mechanism, with an excellent score and its discovered interaction network (Fig. 8) [92], plausibly explains its activity.

#### Antituberculosis therapy

The WHO launched the"END TB"plan in 2014 with the goal of reducing TB fatalities by 95% and TB incidence by 90% by 2035 as compared to 2015 levels [93]. The number of Mycobacterium tuberculosis (*M. TB*) infections that are resistant to the most effective medications, such as isoniazid [INH] and rifampicin [RIF], has been steadily rising over the last ten years. In 2018, 484,000 people worldwide developed drug-resistant tuberculosis [94]. Current first-line treatments for tuberculosis is administration of a combination of INH, RIF, pyrazinamide (PZA), and ethambutol (EMB) for the first two months. This is followed by a prolonged course of INH and RIF for an additional four to seven months. But there are now major worries about the rise and dissemination of extensively drug-resistant (XDR) and multidrug-resistant (MDR) strains of *M. tuberculosis* [95]. It has been claimed that a number of new medications, such as bedaquiline, delamanid, and pretomanid, have been developed [96–98]. However, these medications have already demonstrated resistance and failure in ordinary clinical practice [99]. Therefore, there is an urgent need for research into new anti-TB drugs.

A carbapenem antibiotic called biapenem **5** has been studied in clinical trials to treat strains of *Pseudomonas aeruginosa* [100]. Additionally, tebipenem **6** was the first carbapenem to be taken orally for the treatment of bacterial pneumonia, respiratory infections, and otitis [101]. When Kaushik et al*.* evaluated the efficacy of carbapenems against *M. tuberculosis* in 2015, they showed that biapenem **5** and tebipenem **6** had bactericidal action against *M. tuberculosis* H37Rv in vitro [102].

**Fig. 9:**
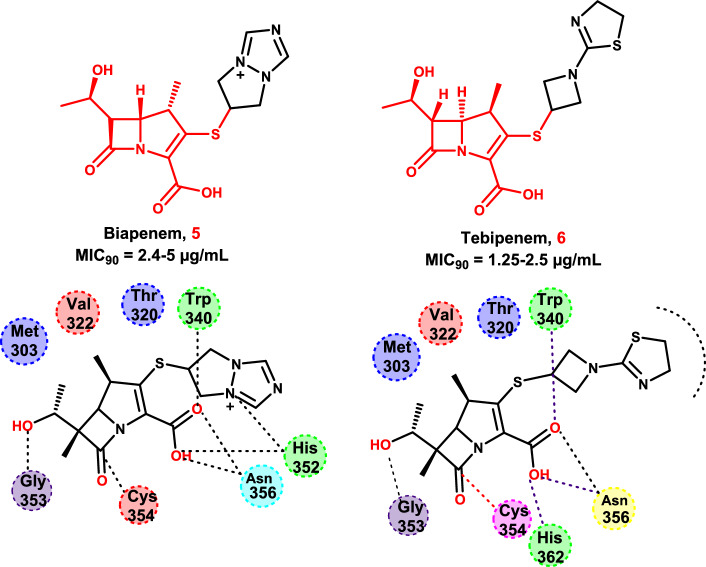
The docking results of Biapenem **5** and Tebipenem **6** to the outer cavity of LdtMt2 (PDB codes 5DCC and 5DC2, respectively)

In 2017, Kumar and his team members conducted an investigation into how carbapenems, such as biapenem **5**, tebipenem **6**, faropenem, and doripenem, suppress L, D-transpeptidase-2 (LdtMt2), thereby producing growth inhibition activities against *M. tuberculosis* [103, 104] (Fig. 9).

The carbapenem carboxylate of biapenem **5** and tebipenem **6**, accepts hydrogen bonds from **His352**, **Asn356**, and **Trp340**. The main-chain NH groups of **His352**, **Gly353,** and **Cys354** surround the carbonyl oxygen of the β-lactam ring. Biapenem and tebipenem form a thioester bond with the catalytic residue of LdtMt2 (**Cys354**) and extend into the outer cavity (Fig. 9) [104, 105].

**Fig. 10:**
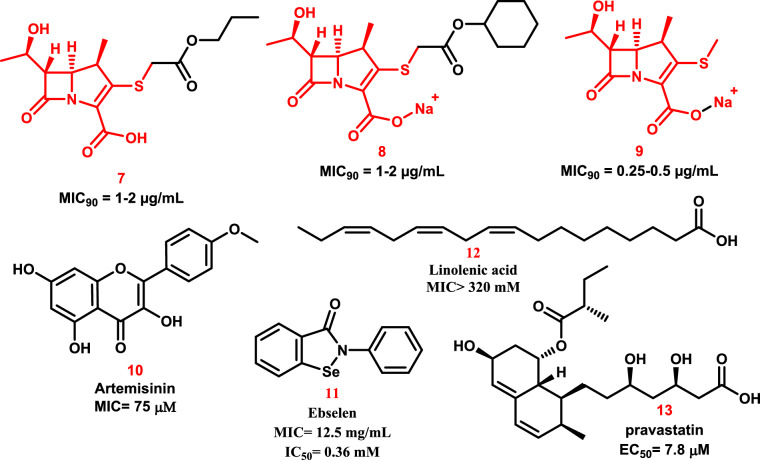
The molecular structures of the different repurposed and discovered anti-TB agents

In light of this finding, they created and synthesized thirteen carbapenems with enhanced anti-TB properties [103]. Three of the novel carbapenems produced the lowest MICs against *M. tuberculosis*: compounds **7** and **8** had MICs of 1–2 μg/mL, while compound **9** had a MIC of 0.25–0.5 μg/mL (Fig. 10).

Kumar et al*.* demonstrated that the pyrrolidine carboxyl group and substituent primarily engage with the outer cavity within a hydrophilic cleft that includes the"oxyanion hole"(including Asn356, His352, and Trp340) and establish a large number of hydrogen bonds with different LdtMt2 residues. It was demonstrated that the alkyl tails of compounds 7, 8, and 9 formed hydrophobic contacts with Trp340 in the outer cavity [103].

Zheng et al*.* screened a small-molecule library of about 540,000 compounds using a DosRST-dependent fluorescent *M. tuberculosis* reporter strain. They found that artemisinin **10** is a new compound that blocks the DosRST regulon [106, 107]. This stops the physiological processes that are linked to *M. tuberculosis* persistence. The DosS and DosT histidine kinases carry the heme sensor, which artemisinin **10** specifically targets to alter the DosRST signaling pathway (Fig. 10) [108, 109].

Ebselen **11** (Fig. 10) is a selenium-organic, non-toxic substance that has cytoprotective, anti-inflammatory, antioxidant, and anti-atherosclerotic characteristics [110]. Proposals suggest that Ebselen inhibits *M. tuberculosis* and methicillin-resistant *Staphylococcus aureus* [111, 112]. Several genome sequencing studies done by Padiadpu et al. in 2016 showed that ebselen and INH work very well together to fight *M. tuberculosis* H37Rv and three clinically drug-resistant strains (JAL2287, BND320, and MYC431) [113]. In 2017, Goins and colleagues published a crystal structure showing that ebselen derivatives covalently alter *M. tuberculosis*’ Cys209 residue, of *M. tuberculosis*, forming a selenenylesulfide bond and stabilizing antigen 85 C (Ag85C). On the other hand, the molecular docking study of de Munnik et al*.* showed that the benzisoselenazolone ring could be opened by changing the cysteine to Cys354. This created a complex that had a lot of hydrophobic interactions with the active-site lid of Ldt_Mt2_ [114].

One of the primary components of human cell membranes is linolenic acid (LNA) 12 (Fig. 10), an omega-3 polyunsaturated fatty acid (PUFA) that is present in dark green plants as glycerides. The body can produce, metabolize, and transform this PUFA [100]. In 2016, resazurin microtiter and MGIT 960 system assays were used to establish the dose-dependent anti-TB properties of linolenic acid **(12)** and conjugated-linoleic acid (CLA) [115]. It’s interesting to note that *M. tuberculosis* growth and proliferation were successfully reduced by a 21-h treatment with either LNA or CLA, with MICs of 200 mg/mL. These findings suggest that LNA and CLA’s strong anti-TB properties entail a novel pharmacological activity or action, leading to a targeted anti-TB impact. As a result, these PUFAs are viable options for strong anti-TB medications.

A competitive inhibitor of HMG-CoA reductase, pravastatin (Fig. 10) is used to treat hypocholesterolemia [116]. Pravastatin **(13)** has positive benefits against *M. tuberculosis*, according to Dutta et al*.*, with a half-maximal effective concentration (EC_50_) of 7.8 µM in 2019. Additionally, they discovered that pravastatin caused a differential augmentation of INH, RIF, and Pyrazinamide’s (PZA) antitubercular actions. Researchers have demonstrated that pravastatin **13** dramatically reduces the lung bacillary burden in a mouse model of human-like necrotic TB lung granulomas. When looked at together, these results strongly support the benefits of pravastatin’s clinical testing as an extra treatment for tuberculosis that targets the host [117].

#### COVID-19 treatment

The coronavirus disease-2019 (COVID-19) pandemic started in December 2019. It has emerged as the world’s most pressing public health issue. The coronavirus family of single-stranded RNA viruses, which includes the severe acute respiratory syndrome coronavirus type 2 (SARS-CoV-2), is the cause of COVID-19. The spatial conformation of the"spike"protein determines the coronal shape of this virus [118].

**Fig. 11:**
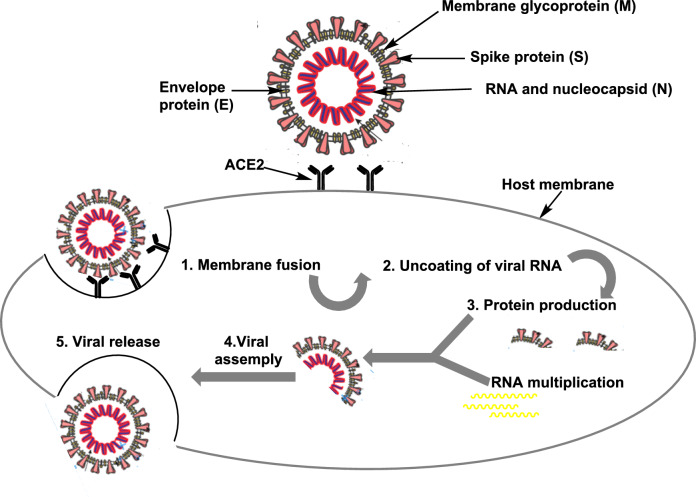
Mode of SARS-CoV-2 infection and multiplication

There are four main structural proteins in SARS-CoV-2. They are the spike (S) protein, the nucleocapsid (N) protein, the membrane (M) protein, and the envelope (E) protein. Each one does different things (Fig. 11) [119]. A map of the protein interactions between SARS and CoV-2 identifies potential therapeutic repurposing targets.

The spike protein of SARS-CoV-2 binds to a receptor known as the angiotensin-converting enzyme 2 (ACE2), a protein present on the surface of some human cells in the gastrointestinal system, lungs, and nasal passages, among other places, to infect a human host [120]. Notably, an enzyme on the host cell’s surface known as transmembrane serine protease 2 (TMPRSS2) is required to split the spike protein before it can enter the host cell.

High levels of tumor necrosis factor (TNF), interleukin-6 (IL-6), interleukin-1β (IL-1β), and C-reactive protein (CRP) are signs of inflammation that happen when ACE2 boosts the immune system, leading to inflammation in the lungs (Fig. 11) [121]. SARS-CoV-2 produces millions of copies of itself after infecting human cells, which individuals can exhale or cough out to infect others.

Few medications have been shown to be effective against SARS-CoV-2 to date. Researchers have linked severe illness and death to pre-existing conditions such as diabetes mellitus [122], chronic obstructive pulmonary disease [123, 124], hypertension, heart and renal illnesses [125], cancer [126], and polycystic ovary syndrome [127]. Several studies [128, 129] indicate that a small number of individuals receiving medication for chronic illnesses do not carry the SARS-CoV-2 infection.

The majority of patients at the start of the pandemic had severe COVID, and not all medication candidates worked for these individuals at the tested dosages. Several medications play distinct roles, including antivirals, immunological boosters, anti-inflammatory agents, immunomodulators, antiparasitic agents, and endocrine system medications. The trials have revealed other potential pharmacological effects. Researchers have identified numerous medications with various modes of action as viable options for treating SARS-CoV-2 patients. Most of the ways that SARS-CoV-2 is stopped involve reducing the damage caused by the inflammatory cytokine storm and stopping virus replication in several different ways [130].

##### Antiviral therapy

Remdesivir **14** can combat a variety of RNA viruses in vitro, including Ebola, and may be useful in the prevention and management of coronavirus infections [131]. It works by preventing viral RNA-dependent RNA polymerase from accomplishing its function. This results in viral exoribonuclease evading proofreading, which drastically reduces the amount of viral RNA produced [132]. The first COVID-19 infection case in the United States received remdesivir treatment, and the patient made a full recovery after just one day. The Food and Drug Administration (USFDA) granted remdesivir emergency use approval for patients hospitalized with severe COVID-19 on May 1, 2020. This final approval was issued due to preliminary evidence of remdesivir’s effectiveness in these patients. Remdesivir treatment by itself is probably insufficient; nevertheless, considering the significant death rate despite its use [133].

**Fig. 12:**
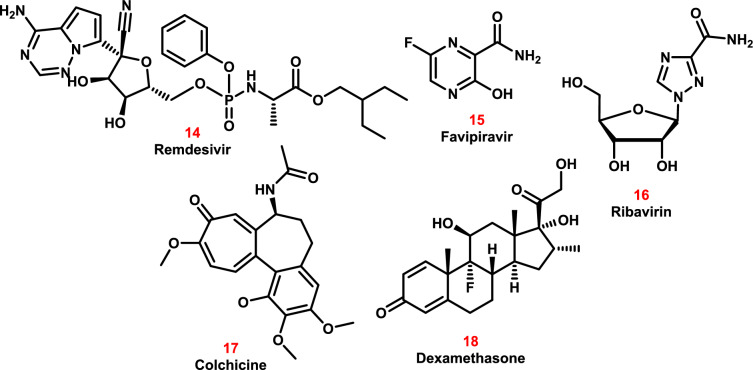
The molecular structures of various antiviral and anti-inflammatory drugs have been repurposed for COVID-19 treatment

An antiviral medication called favipiravir **15** [134] efficiently and specifically inhibits RNA-dependent RNA polymerase (RdRp) in RNA viruses. The medication cures severe fever with thrombocytopenia syndrome, Lassa fever, rabies, and Ebola virus disease, among other potentially fatal human infections. Compared to other viral RdRps, SARS-CoV-2’s RdRp activity was ten times more [135, 136]. Furthermore, favipiravir’s mode of action is distinct from other anti-influenza medications in that it directly inhibits viral transcription and replication. Given that human cells lack the RdRp structural domain, favipiravir’s broad-spectrum coverage makes it a strong contender (Fig. 12).

The lowest toxicity and adequate antiviral activity were reported in vitro at an ideal dosage of 2 mg/mL [137]. In vivo research showed that high doses of favipiravir greatly reduced the amount of SARS-CoV-2 in the lungs of hamsters infected with the virus and improved the histopathology of the lungs. The study found that favipiravir was safe and worked better than controls at shortening the time that SARS-CoV-2 RNA was shed in patients who had a recurrence after being discharged [138]. The Central Drugs Standard Control Organization has authorized favipiravir for COVID-19 for sale after completing phase IV clinical studies.

A synthetic guanosine nucleoside called ribavirin (RV) **16** is used to treat some types of hepatitis C. RV disrupts the production of viral mRNA and exhibits broad-spectrum action against a variety of RNA and DNA viruses. In the early phases of SARS-CoV-2 pathogenesis, clinical trials show that RV has a considerable therapeutic effect (Fig. 12) [139, 140].

Currently, numerous clinical trials have shown no significant adverse effects. Similar to the 500 mg/day amount used to treat SARS-CoV-2, the same dosage is used to treat viral hepatitis C. Additionally, 450 mg/day is used to treat viral respiratory tract infections.

##### Anti-inflammatory drugs and immunotherapy

The alkaloid Colchicine **17** alleviates the excruciating pain of gout attacks and treats the inflammatory symptoms of familial Mediterranean fever, an inherited auto-inflammatory condition (Fig. 12) [141, 142]. SARS-CoV-2 starts the inflammatory process by turning on the NLRP3 (nucleotide-binding domain, leucine-rich–containing family, pyrin domain–containing–3) inflammasome, which releases a storm of cytokines. To lessen excessive inflammation, colchicine can target NLRP3 inflammatory vesicles.

High dosages of colchicine have been shown in available clinical trials to considerably improve or clear SARS-CoV-2 [143, 144].

Dexamethasone **18**, a glucocorticoid with strong anti-inflammatory qualities, treats a number of inflammatory conditions, including rheumatoid arthritis, endocrine disorders, and bronchial asthma. In addition to binding to certain nuclear steroid receptors, dexamethasone stops the activation and death pathways of NF-kB (nuclear factor kappa-light-chain-enhancer of activated B cells). While greater dosages of corticosteroids have immunosuppressive effects, lower doses have anti-inflammatory effects and stimulate anti-inflammatory genes, like IL-10 (Fig. 12) [145].

In a multicenter randomized controlled trial conducted in Spain [146], it was shown that early dexamethasone administration decreased overall mortality and the length of time patients with established moderate-to-severe COVID-19.

##### Selective estrogen receptor modulator

According to COVID-19 pandemic data, men were more likely than women to experience problems from SARS-CoV-2 infection, and middle-aged and older men were particularly at a much higher risk of dying or suffering from severe disease [147]. Women, particularly those not yet menopausal, received protection [148]. Estrogen is known to have strong immunomodulatory and anti-inflammatory effects on COVID-19 [149, 150]. In addition to decreasing the expression of ACE2 receptors on host alveolar epithelial cells, which stops the SARS-CoV-2 virus from entering host cells, estrogen, which is primarily given subcutaneously, also inhibits viral replication by taking up the active site of the protease TMPRSS2, which is necessary for viral replication. Furthermore, estrogen has antithrombotic, immune cell-regulating, antioxidant, anti-inflammatory, and endothelial cell-protective qualities [151–155].

**Fig. 13:**
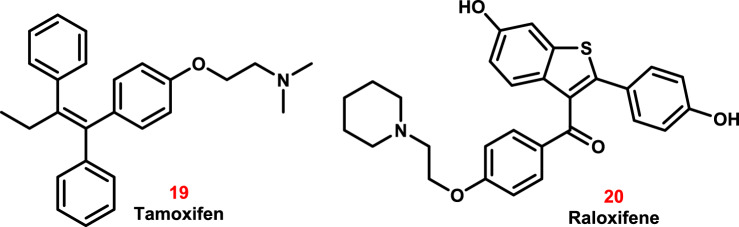
The molecular structures of various SERM drugs have been repurposed for COVID-19 treatment

First-generation selective estrogen receptor modulators (SERMs), like tamoxifen **19**, treat estrogen receptor-positive breast cancer and reduce the risk of breast cancer in high-risk individuals [156]. Researchers discovered its effectiveness against the herpes simplex virus (HSV), hepatitis C virus (HCV), and human immunodeficiency virus (HIV) [156]. Tamoxifen may also change the speed of endosomes and raise the pH of lysosomes, which could stop SARS-CoV-2 from invading (Fig. 13) [157].

Raloxifene **20**, being a second-generation of SERM, binds to estrogen receptors [158]. Raloxifene demonstrates an in vitro antiviral activity, in terms of inhibition of viral replication and/or infection, against influenza A, and HCV. Iaconis et al. [159] claimed that raloxifene is a potential pharmacological agent against SARS-CoV-2 (Fig. 13).

### Cancer therapy

#### Repurposing of the old drug iodoquinol to a series of novel anti-cancer 7-iodo-quinoline-5,8-diones

**Fig. 14:**
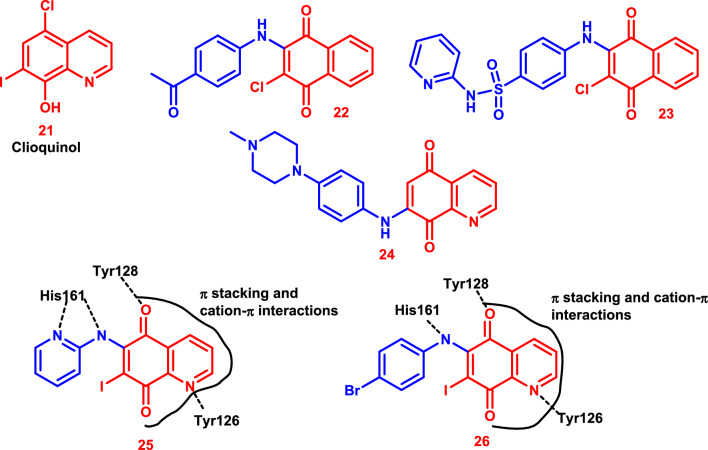
Various examples of quinone-based drugs have been repurposed for cancer treatment

Researchers have identified elevated intracellular reactive oxygen species (ROS) and antioxidant defense mechanisms as one of the characteristics of cancer cells. Cancer cells are more reliant on the"redox adaptation"process and produce more ROS than normal cells to preserve their malignant phenotypes [160–162]. Therefore, malignant cells up-regulate antioxidant enzymes, the most crucial of which is NAD(P)H quinone oxidoreductase (NQO1) [163], to combat the increased intrinsic oxidative stress (Fig. 14).

NQO1 is a protein that uses either NADPH or NADH as a reducing cofactor [164–166] to detoxify the metabolism of endogenous and exogenous quinones to their corresponding hydroquinones by avoiding the generation of harmful superoxide species by two-electron reduction. To protect against oxidative stress brought on by cytotoxic chemicals, NQO1 also functions as an antioxidant enzyme that eliminates superoxide [167]. It is believed to help maintain some endogenous antioxidants in their reduced and active forms, including α-tocopherolquinone and coenzyme Q10 [168].

It also prevents the 20S proteosome from degrading a number of tumor suppressors, including p53, p33ING1b, and p73 [169–171]. Thus, inhibiting NQO1’s protective and chaperone roles will eventually lead to the reduction of tumor growth, which makes this an attractive method to developing powerful cancer chemotherapeutic medicines [172].

As a result, novel anti-tumor medicines were developed by utilizing the concept of repurposing drugs to target NQO1. This was accomplished by using the classic antifungal and antiparasitic medication clioquinol **21** as the study’s first lead structure. Clioquinol **21** has demonstrated anti-cancer efficacy in several in vitro and in vivo experimental models [173, 174]. According to a recent study, it can improve apoptosis and block the NF-κB (Nuclear factor kappa-light-chain-enhancer of activated B cells) signaling pathway, which increases the radiosensitivity of cancer cells (Fig. 14) [175].

Furthermore, it appears that the 1,4-naphthoquinones **22** and **23** (PI-083) primarily prevent cell proliferation by triggering apoptosis [176, 177]. The amino-quinoline-5,8-dione **24** also stopped NQO1 from working, which made it very effective at stopping the growth of drug-sensitive HeLaS3 cells and multidrug-resistant KB-vin cells (Fig. 14) [178].

Thus, effective anti-tumor drugs, 7-iodo-quinoline-5,8-diones **25** and **26**, were investigated by combining pharmacophore hybridization with drug repurposing. Perhaps due to NQO1 inhibition, these compounds inhibit malignant MCF-7 cell lines, resulting in a decrease in NAD levels. They were also able to increase the amount of reactive oxygen species and lower the expression of p53, which makes the NQO-1 inhibitory effect stronger. The 5,8-quinolinedione moiety in both **25** and **26** was involved in π stacking and cation-π interactions with NQO1, and the hydrogen bonds with Tyr126, Tyr128, and His161 were maintained while docking within the active region of NQO1 (Fig. 14).

#### Nitrofurazones and different antimicrobial medications are being repurposed as possible anti-cancer medicines

For almost 70 years, a class of nitro chemicals known as clinical 5-nitrofurans (NFs) has been used to treat bacterial infections [179]. The primary drivers of the NFs’ well-defined antibacterial mechanism are the azo-[180] and nitro-reduction [181] processes, which produce toxic free ROS and create intracellular oxidative stress conditions. These free radicals interact with biological nucleophiles, including proteins, enzymes, and nucleic acids, to kill the bacteria.

**Fig. 15:**
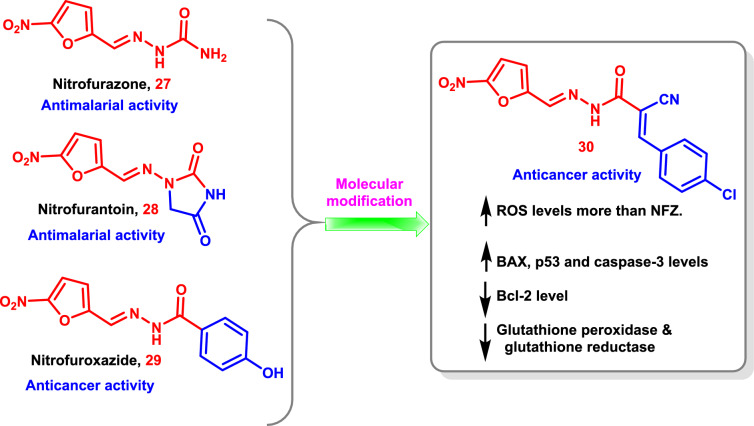
Modification of the reported antimalarial and anti-cancer drugs **27**–**29** to the 5-nitrofurazone-based analog **30** as a potent anti-cancer agent

By blocking the glutathione reductase enzyme, nitrofurazone (NFZ) **27** and nitrofurantoin **28** were successfully repurposed as antimalarial medications [182–184]. Also, by disrupting the mitochondrial ROS pathway, researchers found that nitrofuroxazide **29** produced anti-cancer effects (Fig. 15) [185].

Repurposing the microbial drugs **27** and **28** as potential anti-cancer medications against different cancer cell lines was documented and novel nitrofurazone analogs were designed and created taking into account the significant pharmacologic characteristics present in NFZ (Fig. 15).

Compound **30** was shown to be more active than NFZ in inhibiting the proliferation of all examined cancer cells with observable morphological collapse among the produced analogs. Comprehensive biological investigations have shown that **30** may raise intracellular ROS levels and induce apoptosis by markedly down-regulating Bcl-2 expression and up-regulating BAX, p53, and caspase-3 expression levels more than NFZ. In addition, compound **30** had almost two times as much inhibition of glutathione reductase and 1.5 times as much inhibition of glutathione peroxidase compared to NFZ (Fig. 15) [186].

#### Repurposing of cardiovascular agents to anti-melanoma agents

β-Adrenergic receptor antagonists (β-blockers), a class of medications widely used to treat hypertension, arrhythmia, and angina pectoris, have recently been shown to have anti-cancer properties in vitro, in vivo, and in clinical settings [187–190]. Propranolol **31**, a non-selective β-blocker, has been shown in a number of studies to be effective against a variety of malignancies, including melanoma [176], liver carcinoma [191], breast cancer [192], prostate cancer [193], and non-small cell lung cancer (NSCLC) [194]. Propranolol (80 mg daily) from the time of diagnosis considerably delayed the recurrence of melanoma and lowered the risk for users by almost 80%, according to research published in JAMA Oncology [195]. Additionally, in vitro, propranolol may cause cell arrest, apoptosis, and inhibition of cell growth in a number of melanoma cell lines and patient-derived primary cells [196–198].

**Fig. 16:**
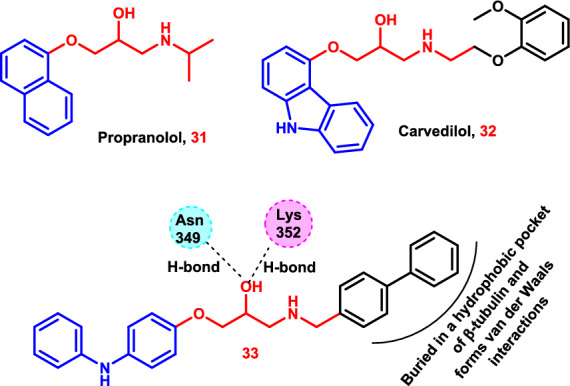
The molecular structures of the candidates **31–33** and the binding mode of **33** into the β-tubulin and α-tubulin interface

The efficacy of the authorized β-blocker carvedilol **32** was assessed using a 3-(4,5-dimethylthiazol-2-yl)−2,5-diphenyltetrazolium bromide (MTT) test on three human malignant melanoma cell lines (SK-MEL-5, SK-MEL-28, and A-375). It showed unexpected IC_50_ values against all examined cell lines, ranging from 13.0 to 15.5 μM (Fig. 16) [199].

Propranolol and carvedilol, two important β-blockers, were utilized to begin structure development and optimization based on preclinical and clinical investigations. This was a step in the therapeutic repurposing and rediscovery process from cardiac drugs to anti-melanoma drugs. Molecular optimization of both propranolol and carvedilol led to the formation of the compound **33**, which showed significant potency against human melanoma cell growth (IC_50_ = 1.98–3.70 µM). Additional biological analysis showed that **33** might cause cell death, hinder colony formation, inhibit cell migration, and cause G2/M phase arrest in melanoma cell lines. Tubulin may be a possible target since it could interfere with the cellular microtubule network (Fig. 16).

A structural understanding of the interaction between tubulin and **33** was provided by molecular docking experiments. The colchicine binding site is the configuration that compound **33** takes between the β-tubulin and α-tubulin heterodimers. The diphenylamine moiety is exposed toward the α-tubulin T5 loop, and the diphenyl moiety forms van der Waals interactions with tubulin. The diphenyl moiety is buried in a hydrophobic pocket of β-tubulin, encircled by strands S8, helix H7, and T7 loop. The hydroxyl in the propan-2-ol linker makes an H-bond with the Val351 side chain, whereas the amine *N* atom forms an H-bond with the Lys352 and Asn349 side chains. The tight binding into the β-tubulin and α-tubulin interface, particularly the β-tubulin domain, may be facilitated by these H-bonding interactions (Fig. 16) [200].

#### Repurposing of antidepressant drugs to anti-cancer agents

Preclinical research revealed that certain antidepressant medications exhibited anti-cancer potential through various pharmacological processes related to tumor immunology, oxidative stress, cell proliferation, metastasis, cell cycle, apoptosis, and autophagy [201]. Clinical trials for the treatment of cancer have been initiated for three licensed antidepressants: tranylcypromine, phenelzine, and desipramine (TCP) [202, 203]. Based on the popular antidepressant TCP, several LSD1 (Lysine-specific Histone Demethylase 1 A) inhibitors have shown promising clinical therapeutic promise [204].

**Fig. 17:**
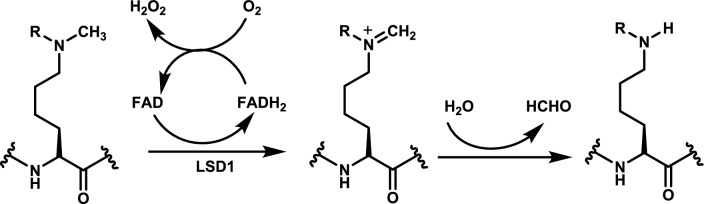
The mechanism of demethylation of histone H3K4me1/2 H3K4 to H3K4 methyl eraser by LSD1

LSD1 was the first to be recognized, sharing a conserved catalytic domain with MAOs (monoamine oxidases) (17.6% of structural homology) and lysine-specific demethylase 2 (LSD2, 45% of structural homology) [204]. LSD1 can selectively demethylate mono- and di-methylated lysine residues of histone H3K4me1/2 to serve as an H3K4 methyl eraser (Fig. 17).

According to clinical research, LSD1 overexpression has been linked to the emergence of a number of malignancies, making it a potential target for anti-cancer medications [204, 205]. Because LSD1 and MAOs share similar sequences, structures, and catalytic mechanisms, medications that target MAOs may also inhibit LSD1. Research revealed that TCP had strong inhibitory effects on LSD1, MAO A, MAO B, and LSD2 [204, 205].

**Fig. 18:**
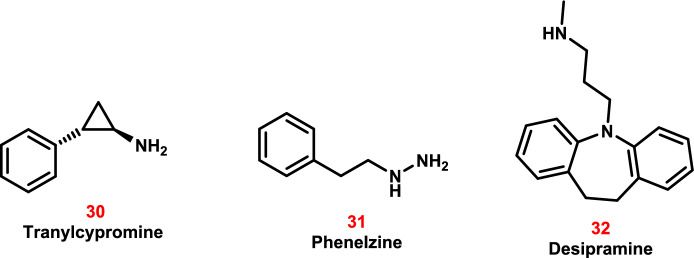
The molecular structures of the antidepressant drugs **30–32**, which were repurposed as anti-cancer candidates

Tranylcypromine (TCP, Parnate, **34**) was first discovered in the 1940 s as an amphetamine analog to cure nasal congestion [206]. Further clinical research revealed that TCP elevated neurotransmitter levels in the brain by restricting serotonin and norepinephrine catabolism, and the US Food and Drug Administration (FDA) approved it as an antidepressant drug in 1961 for patients who suffered from major depressive disorder [206]. TCP also demonstrated anti-tumor activity by targeting LSD1 (Fig. 18).

Aside from its role in neurotransmitter metabolism, TCP demonstrated unique biological functions at low doses by suppressing drug-metabolizing enzymes such as CYP2A6 and CYP2C19. This action may result in pharmacokinetic drug interactions [206]. Combination therapy of **TCP** and all-*trans*-retinoic acid (ATRA, tretinoin) resulted in more significant anti-leukemic activity compared with each drug alone, as shown by Schenk et al. This combination therapy, which inhibits ATRA’s normal pro-differentiation role, paved the way for acute myeloid leukemia (AML) treatment (Fig. 18) [207].

Phenelzine (Nardil, **35**) is a clinically used antidepressant that works by rebalancing neurotransmitters in the brain. Preclinical studies have shown that phenelzine suppresses prostate tumorigenesis [208]. A Phase II clinical trial (ClinicalTrials.gov Identifier: NCT02217709) was launched to assess the therapeutic efficacy of phenelzine in prostate cancer patients. Furthermore, phenelzine analogs were shown to be effective in modulating histone methylation in cancer cells by targeting LSD1 [209].

Desipramine (Norpramin, **36**) is an antidepressant drug that is clinically used as an adjunctive treatment for cancer patients. Pathological examination revealed that it inhibited the proliferation of HCC Hep3B cells by promoting apoptosis, triggering MAPK signaling, and elevating intracellular Ca^2+^ levels [210]. Desipramine also promoted death in human cancerous prostate PC-3 cells by stimulating the JNK kinase and caspase-3 networks and elevating Ca^2+^ levels (Fig. 18) [211, 212].

#### Repurposing of ascorbic acid as an anti-cancer agent

**Fig. 19:**
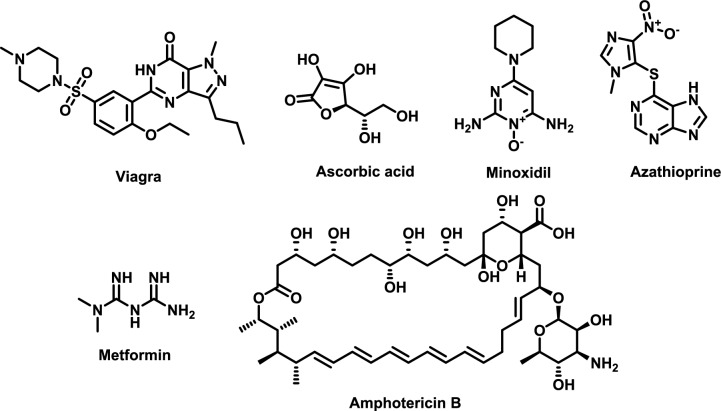
The molecular structures of miscellaneous cases of successful drug repurposing

Ascorbic acid, commonly known as vitamin C, is an essential nutrient for humans and has been consistently included in the human diet. It cannot be synthesized by the human body, making it a vital micronutrient for survival [213]. The growth and development of the human body rely entirely on adequate intake of ascorbic acid, which is necessary for the production of connective tissue, the formation of blood clots, the synthesis of catecholamines, and the antioxidation of vitamin E [214]. Additionally, it facilitates the absorption of iron from dietary sources, serves as a reducing agent in the citric acid cycle, and significantly contributes to the synthesis of carnitine, steroids, and neuropeptides [215]. It plays a critical role in numerous biochemical functions, including neurotransmission in the brain and maintaining the brain’s structure. Earlier reports suggest that intravenous administration of pharmacologic doses of ascorbic acid generates hydrogen peroxide, which may selectively damage cancer cells (Fig. 19) [216, 217].

#### Repurposing of Metformin as an anti-cancer agent

Metformin, one of the oldest and most studied drugs, is the first-line treatment for type 2 diabetes [218]. The Food and Drug Administration (FDA) approved metformin in 1994 [219]. Metformin also has several non-FDA-approved uses, including preventing type 2 diabetes, treating and preventing polycystic ovarian syndrome (PCOS), and controlling weight gain caused by antipsychotics [219]. To determine whether metformin can reduce the risk of cancer in people with type 2 diabetes, researchers are looking into the medication’s possible anti-aging, anti-cancer, and neuroprotective qualities [220]. These include cancers of the breast, colon, and prostate.

Beyond glycemic control, metformin has several applications in prediabetes and gestational diabetes, as well as ongoing research into its potential for cancer risk reduction, enhancement of cognitive function, and possible anti-aging effects.

Metformin has demonstrated an increasing ability to inhibit the development, survival, and spread of various types of tumor cells, including malignancies of the breast, liver, bone, pancreas, endometrium, colon, kidney, and lung [220]. In particular, metformin inhibits the mammalian target of rapamycin (mTOR) signaling by activating AMPK. Consequently, protein synthesis is disrupted, which hinders cell division and growth. Interestingly, metformin may interfere with the communication between insulin receptor signaling systems and G protein-coupled receptors (GPCRs), which could aid in preventing the spread of pancreatic cancer [221].

By modifying the immune response within the tumor microenvironment, metformin shows promise as an adjuvant medication for cancer treatment, as suggested by both preclinical and clinical research [222]. Key processes involved include reduced production of reactive oxygen species (ROS), decreased DNA damage, enhanced autophagy, increased apoptosis, activation of p53, inhibition of mTOR signaling, and a diminished inflammatory response (Fig. 19).

#### Repurposing of aspirin as an anti-cancer drug

Studies have explored aspirin’s potential to prevent various cancers, including colorectal, breast, and prostate cancer [223]. Researchers have recently investigated the relationship between aspirin consumption and chemotherapy medications. Research has shown that aspirin amplifies the anti-cancer effects of doxorubicin in breast cancer. Doxorubicin and aspirin combined induce apoptosis and decrease the growth of MCF-7 breast cancer cells [224, 225]. By inhibiting the MEK/ERK signaling pathway, aspirin may also increase the sensitivity of breast cancer cells to doxorubicin. The combined use of both medications alters the mitochondrial membrane potential, resulting in increased calcium influx into the cell, the release of cytochrome C and apoptosis inducing factor (AIF) proteins, and the initiation of cell death through caspase activation [226].

As the first non-steroidal analgesic and anti-inflammatory medication, aspirin (ASA) was initially made available in powder form by Bayer & Co. in 1899. It had been noted clinically in 1948 that 400 of patients who received aspirin did not have a heart attack [227]. ASA’s antiplatelet activity was later identified as the mechanism behind this preventative effect. This was the first time a medication had been repurposed.

Although ASA selectively inhibits COX-1 at low concentrations, it also acetylates COX-2 and various other proteins and nucleic acids non-specifically at high concentrations and following extended use. The effectiveness of aspirin in preventing myocardial infarction in individuals with cardiovascular risk factors was supported by its antiplatelet properties [228].

The only non-steroidal anti-inflammatory medication (NSAID) that can acetylate and permanently deactivate both COX-1 and COX-2 isoforms through covalent contact is aspirin. COX-2 is produced in cells by activation of growth factors and inflammatory cytokines, whereas COX-1 is broadly dispersed in tissues such as the stomach mucosa and platelets [228].

Aspirin binds to Ser 516 in the active site of COX-2 in the same way as it binds to Ser 530 in the active site of COX-1. Accordingly, inhibition of COX-1 predominantly leads to antiplatelet action [229]. Whereas suppression of COX-2 adds to anti-inflammatory effects. Low-dose aspirin (75–80 mg) is beneficial in decreasing platelet aggregation. However, a greater dose (> 325 mg) is required for anti-inflammatory activity. This is partially because it is a substantially more powerful inhibitor of COX-1 (0.3 µg/ml) as opposed to COX-2 (50 µg/ml) (5). So, at the anti-inflammatory dose, it also irreversibly inhibits COX-1 in the gastric mucosa, leading to ulceration [229].

## Miscellaneous cases of successful drug repurposing

### Viagra

The class of drugs known as phosphodiesterase inhibitors includes sildenafil, better known as the prescription medication Viagra [230]. Created as an inhaled medicine to quickly relieve angina pectoris, tests in men indicated that sildenafil enhanced penile erections, leading to a shift in focus toward treating impotence. This change opened new avenues for research and development in erectile dysfunction treatments [231]. As a result, sildenafil became one of the most recognized and widely prescribed medications for this condition, significantly impacting the lives of many individuals. Patients with erectile dysfunction viewed this activity as a repurposing opportunity due to the limitations of existing treatments [232]. Consequently, the use of sildenafil also extends to the treatment of high-altitude pulmonary edema, idiopathic pulmonary fibrosis, and other rare conditions [233].

Sildenafil (Fig. 19) inhibits the breakdown of cGMP in the corpus cavernosum by blocking cGMP-specific phosphodiesterase type V, which facilitates the relaxation of smooth muscle and enhances blood flow, resulting in an erection. cGMP also relaxes smooth muscle and increases blood inflow, contributing to the development of an erection [234]. At elevated levels, sildenafil reduces body fat and improves the biochemical markers associated with metabolic syndrome in test subjects [235]. Additionally, sildenafil has been shown to have rapid antidepressant effects and to activate autophagy, a process in which cellular cytoplasmic components and intracellular organelles are chemically tagged and digested by intracellular enzymes [236]. However, further research is required to evaluate the advantages and disadvantages of sildenafil treatment [237].

### Minoxidil

Minoxidil (MXD) is an antihypertensive medication acting by peripheral vasodilation [238]. Researchers noticed that some patients taking it experienced increased hair growth as a side effect. Rogaine (brand name MXD) was approved by the Food and Drug Administration in 1988; it was the first drug to win the agency’s endorsement for male pattern baldness. Studies indicated that men on the medication, which was applied directly to the scalp, had a slower rate of hair loss and, in some cases, regrew hair. In 1991, the FDA approved a lower dose for women and aggressively marketed the brand as an over-the-counter medication.

Its mechanism of action involves potassium channel opening and increasing blood flow into hair follicles by releasing nitric oxide [239]. MXD helps hair grow by extending the growth phase of hair follicles and increasing a substance called vascular endothelial growth factor (VEGF), which promotes hair growth. Recent studies showed the combination therapy of MXD and platelet-rich plasma (PRP) was superior to monotherapy for either treatment (Fig. 19) [240].

### Azathioprine

The immunosuppressive medication azathioprine was initially prescribed to prevent transplant rejection. It continues to be commonly used to treat approximately one hundred autoimmune disorders. It has been suggested as a possible treatment for severe COVID-19 cases because it can lower the levels of IL-6 and IL-7, which are high in these situations. Therefore, its role in mitigating the cytokine storm has suggested it as a possible treatment for severe COVID-19 cases [241]. To prevent transplant rejection, azathioprine can be administered alone or in combination with other immunosuppressive drugs.

Azathioprine (Fig. 19) is effective for treating conditions that rely on steroids and do not improve with high doses of immunosuppressive drugs like methylprednisolone. Several diseases, including rheumatoid arthritis, inflammatory bowel disease, and systemic lupus erythematosus, can be managed with azathioprine. However, azathioprine is not suitable for the rapid improvement of these conditions, as it may take up to 12 weeks to observe a response. The goals of treatment are effective symptom reduction and the preservation of the patient’s quality of life. Treatment can only alleviate these symptoms as long as taking azathioprine; it does not provide a cure [243].

### Amphotericin B

The mpox virus (MPXV) is the causative agent of monkeypox, often known as mpox. A multi-country Mpox outbreak in 2022 has raised serious concerns due to the disease’s rapid spread. Limited evidence supports the effectiveness of vaccines based on the *Vaccinia virus* (VACV) against the 2022 Mpox outbreak, although they have been demonstrated to elicit a cross-reactive and protective immune response against MPXV. Moreover, Mpox does not have a specific antiviral medication. Several viruses have found that host-cell lipid rafts—small, extremely dynamic plasma-membrane microdomains rich in phospholipids, glycosphingolipids, and cholesterol—are essential surface-entry platforms [244].

Amphotericin B (AmphB) is a macrolide, polyene antibiotic that has a low propensity for drug resistance and a wide range of efficacy against molds, yeasts, and the protozoan parasite Leishmania spp. [245]. Compared to other antifungal medications, resistance to AmB is still uncommon. This is probably because, in contrast to other antifungals that target an enzyme, AmphB targets a significant component of the cell membrane.

It has been documented that Amphotericin B (AmphB) prevents fungal, bacterial, and viral infection of host cells by trapping host-cell cholesterol and interfering with lipid raft formation. In this way, the idea was explored that AmphB could be a new or extra treatment for human Mpox by stopping MPXV from infecting host cells by breaking apart lipid rafts and changing the position of receptors/co-receptors that help the virus entery (Fig. 19) [245].

## Challenges and limitations in drug repurposing

Drug repurposing has become a popular tactic with significant benefits over traditional methods as interest in drug creation at lower production costs has grown. However, investigating novel therapeutic options is not always simple and occasionally hides the significant difficulties that come with this kind of strategy. A repurposed drug should have more controllable safety and regulatory risks and have the required technologies, expertise, and resources. It also has the unique benefit of speeding up the drug discovery process, which includes lower costs, shorter development times, and most importantly, lower failure rates.

As a result, there is now a wealth of evidence in scientific studies demonstrating the connection between drugs and diseases, along with an increase in available chemical and biological resources and reduced storage costs. These factors have contributed to significant growth in the development of drug repurposing projects. It should be emphasized that identifying crucial biological links and integrating fragmented, heterogeneous data from various fields may pose additional challenges not yet addressed in other drug discovery methods. To support decision-making, it is essential to transform this data into relevant, useful, and collective insights. Thus, computational repurposing has focused on combining and evaluating diverse types of information for the same medication, allowing for complexity to be presented clearly and concisely. Furthermore, network-based representations may connect the gathered evidence to significant clinical signatures. These illustrations may also underscore the existing linkages between repurposing scenarios and offer new perspectives to guide the drug discovery process.

## Future perspectives

With strong patient privacy, data security, governance frameworks, and ethical safeguards in place, drug repurposing has recently increased, utilizing large amounts of patient health data from health systems, integrated delivery networks, and clinical data-driven businesses. It is possible to create cancer-ready assays and a diverse range of medications for every target that tests positive. We should be prepared to respond to viral threats through internal readiness. The most effective use of pandemic resources may be influenced by phylogenetic biosimilar processes and comparable disease risks. Their expertise may also strengthen industrial research, international networks, and the academic health system.

Investigating the usage of novel combinations of tested medications that show promise involves aspects such as alternative biomodels, three-dimensional modifications, and increasingly human microorganisms. The establishment of a foundation for non-profit licenses is a positive step for the future of older drug licenses. A compilation of information regarding approved medications, human proteins impacted by small molecules, and the treatment activities necessary to study a complex computer system designed for finding treatments inspired the Final Program Review Determination FPRD. To create simpler pharmacological points with a therapeutic proposition and subject-specific databases, the database was constructed, restructured, and categorized using internal, experimental, or theoretical evidence evaluated for associations between proteins and drug usage activities.

## Conclusion and summary

In conclusion, modern drug discovery has garnered attention for its ability to produce drugs rapidly, thanks to the continuously expanding databases of drug candidates and chemicals. This advancement has facilitated the selection of available compounds, especially in the realm of personalized medicine. This review highlights the features and concepts of drug discovery and repurposing research within the scientific community. Furthermore, based on the data collected for this analysis, we believe that innovations in drug repurposing may provide a viable pathway for developing novel applications for medications and substances that have received clinical approval. In terms of effectively linking safety and efficacy data among humans, utilizing an established medicinal chemical candidate has often proven more advantageous than employing a novel candidate. As a result, we believe that our review will assist researchers in exploring new or alternative ways to utilize the therapeutic agents currently available.

## Data Availability

No datasets were generated or analyzed during the current study.
